# Inside, outside and in‐between: The process and impact of co‐producing knowledge about autism in a UK Somali community

**DOI:** 10.1111/hex.12939

**Published:** 2019-07-18

**Authors:** Nura O. Aabe, Fiona Fox, Dheeraj Rai, Sabi Redwood

**Affiliations:** ^1^ Autism Independence Bristol UK; ^2^ National Institute for Health Research Collaboration for Leadership in Applied Health Research and Care West (NIHR CLAHRC West) and Bristol Medical School University of Bristol Bristol UK; ^3^ Population Health Sciences, Bristol Medical School University of Bristol Bristol UK

**Keywords:** autism, community, co‐production, co‐researchers, impact, knowledge, participatory, qualitative, research, Somali

## Abstract

**Background:**

Co‐production is predicated on equal power‐sharing and responsibility in research partnerships. However, relatively few accounts exist that explore the subjective experience of how co‐researchers achieve such equality, from the perspectives of public contributors and researchers.

**Aim:**

This paper aims to provide a unique insight into the process of co‐production, by weaving personal reflections with principles to evaluate the impact arising from co‐produced knowledge. It is based upon participatory research that was initiated by a ‘lay’ person, on behalf of a community organization, seeking support for Somali families who are affected by autism. The paper explores the evolving partnerships that began with community theatre and qualitative research and leading to extensive dissemination and impact, all of which has been jointly owned and negotiated by the co‐researchers and community organizations.

**Discussion:**

Initially, this paper reflects on the process, drawing on principles defined for co‐production in health research and combining it with the co‐researcher's personal reflections of their experiences as insiders and outsiders, stepping in and out of each other's worlds. The value of reciprocity, flexibility and continuous reflection is illustrated. The latter part of the paper explores the impact of this co‐produced knowledge using a theoretical framework, to assess the specific impacts and its broader transformative potential. It demonstrates how (1) opportunities for all partners to be equitably involved to the maximum degree possible throughout the research process can affect social change and (2) co‐produced research can become a catalyst that is dynamic and complex, achieving multi‐layered impact.

## 1 INTRODUCTION

Collaborative models of research are rooted in long‐standing political, social and artistic traditions, and the multitude of collaborative research practices and co‐production models reflects different motivations, activities and discourses.^1^, ^2^ For this reason, coproduction is a contested concept^3^ and has been described as a ‘poorly formulated’ term.^4^, ^5^ Facer and Enwright argue that no single research method can be labelled ‘co‐production’ but ‘Instead, there are myriad different forms, practices and methods that project teams are using to address the question of how to create new knowledge and practice about “communities”’.^5^

In this paper, the authors refer to co‐production as a principle of engaging and integrating the multiple perspectives of stakeholders to shape the understanding, and processes of knowledge generation, its application and use.^6^ This approach – which goes beyond participation and engagement of the public – has been defined as one in which ‘researchers, practitioners and the public work together, sharing power and responsibility from the start to the end of the project, including the generation of knowledge’.^2^, ^7^ Crucially, co‐production is predicated on the sharing of power to create quality services, programmes and policies.^8^ Despite the value placed on equal research partnerships, Banks and colleagues found that ‘there are relatively few published accounts that combine the perspectives of both parties in reflecting on their experiences of the process of collaboration’.^9^ Perhaps rarer still is an account of co‐production that is written primarily from the perspective of the ‘community researcher’. In this paper, we address this gap, with co‐researchers Nura and Fiona sharing reflective insights into their experience of co‐production from inception of the project to the on‐going impact work. By doing so, we illuminate ways to achieve active involvement and equal power‐sharing. This paper responds to recent calls for empirical evidence about the processes and outcomes of co‐production,^1^ highlighting how the co‐researcher's roles and responsibilities affected the process of co‐producing knowledge and how impact was achieved.

This paper is based on a qualitative study which explored the experiences of Somali families who have children with autism, using a community‐based participatory research (CBPR) approach^10^ at all stages of the research. The unique aspect to this study was that the initial idea was raised by Nura, a member of the local Somali community and then developed with the University of Bristol and NIHR CLAHRC West. The research, a relatively small qualitative study, led to extensive dissemination and on‐going impact work, all of which has been jointly owned and negotiated by the co‐researchers and community organizations. The first part of this paper reflects on the process, drawing on principles defined for co‐production in health and mental health research.^7^, ^11^ Throughout their journey, both co‐researchers experienced aspects of the research process as insiders and outsiders, respectively, and they reflect on how they negotiated and renegotiated their roles at every stage. The latter part of the paper explores the impact of this co‐produced research, using a theoretical framework developed by Beckett et al^12^ to assess both the specific impacts and its broader ‘transformative potential’.

## 2 BACKGROUND TO THE PROJECT

It is not uncommon for qualitative health research to have its seeds in personal lived experience. Arguably, it is less common for research to be initiated by a member of the public, who is not a researcher. Nura Aabe came to the UK as a child when her family fled the civil war in Somalia. When her first‐born son was diagnosed with autism, she endured many years of personal struggle, as she tried to understand and accept what this meant for him, for her as his mother, the family and wider community (see Box 1). With no Somali word for autism and prevailing cultural stigma around mental health and disability, she moved from initially hiding her son, to reaching into her community and establishing a support network for Somali families, called Autism Independence (AI). Members of AI worked with a community theatre company to develop a play called ‘Yusuf can't talk’ which was performed nationally and internationally. With growing awareness that the Somali community has a high prevalence of autism,^8^, ^9^, ^10^, ^11^, ^12^, ^13^, ^14^, ^15^ Nura contacted Dheeraj Rai, a university researcher and psychiatrist whose research focussed on autism in migrant communities to ask about further research. Through discussion, Nura and Dheeraj submitted an idea for research to the NIHR CLAHRC West, who works with partner organizations including the NHS, local authorities and universities, to conduct applied health research and implement research evidence to improve health and health care. The idea for an exploratory research study that would be co‐produced with the local Somali community was supported by Sabi Redwood a NIHR CLAHRC West Senior Research Fellow and Fiona, a Senior Research Associate. The mutually agreed aims of the research were to develop a clearer and more nuanced understanding of the range of views on and perceptions of (1) autism in the local Somali community, (2) the process through which a child was identified as having autism and (3) the experiences and challenges of accessing and engaging with services, including suggestions about how the process of diagnosis and receiving services could be improved to fit more closely with social and cultural needs.

Box 1 **Nura’s story**

1 Imagine you arrive in the UK at the age of 11 with your family, fleeing civil war in Somalia (1988). Imagine that you start in primary school and this is your first experience of the English language and the fall of snow. Years later when you marry, imagine that you were told that your first‐born son has a condition that affects his ability to communicate and socialize but you have not heard of the name of that condition in your mother tongue before.

My son was the most beautiful child in the world. As he turns a toddler, he started laughing at himself, had little sleep and no eye contact, he observed an object for a long time. My mother told me how intelligent my son was as he takes his time to understand how the world works! At his 2‐year development check the health visitor asked me if he talks and if he uses eye contact? And then she refers him to a local nursery.

And then that day has come, when I had to meet a team of different professionals, with different roles that I never heard of before. I wasn't really sure what the meeting was about although I did get a number of reports in the post that I never really understood. They said ‘autism, he has autism’. ‘I have never heard of it before’ I said, ‘what does it mean and is he mentally ill?’ I was so upset and defensive but most of all hopeless as I only wished I understood what exactly autism meant in Somali. I break the news to my family who thought there was nothing wrong with Zak and that he was healthy. I was told that another member of the family spoke late and so ‘Zak will talk soon’, I should not be worried, ‘what do western doctors know’, ‘don't tell anyone there is something wrong with your child it will bring shame to the family’.

That was not true. He had autism, a neurodevelopmental disorder which comes with a series of impairments. So much time was wasted between the dichotomy of my family and professionals who diagnosed my baby with severe autism. Desperately I started seeking for information, knowledge and education around the subject. Initially my understanding of the condition was a description of mental illness. Mental illness comes with social exclusion and stigma in the Somali culture. I was offered the ‘Early Bird’ support programme course for parents and carers, offering advice and guidance on strategies for dealing with young autistic children. I dropped the course after attending a few sessions, because I found the terminology and lack of autism concept overwhelming. I felt lost and confused about my son's condition.

For the next 5 years I tried to understand autism and how services for autistic people operated. I began to get involved in local events and initiatives about autism and moved away from my social groups that perceived autism negatively. This was a very difficult decision, but I was desperate for hope and help for Zak. Attending conferences and higher education hugely influenced my understanding of how to facilitate his interaction and communication development. Zak started using Makaton sign language and started developing verbal communication.

It was the day I understood my son's autism that I started helping him and began on a journey to help him to have a voice. I saw Zak's progress and I also saw other members of my local community who were hiding their children, who were similar to Zak. I knew I needed to educate and empower my community and so I set up an organisation called Autism Independence (AI), the aim which was to mainly raise awareness of autism in the Somali community and among professionals who were involved in their children's care.

### 2.1 Methods

It was agreed that a qualitative design would enable these issues to be explored through in‐depth interviews. In order to meet this aim, a community‐based participatory research (CBPR) approach^5^ was adopted, which is underpinned by principles of community engagement and empowerment, mutual respect and co‐learning, as well as commitments to action and improvement. Together Nura and Fiona conducted in‐depth bilingual interviews with 15 Somali parents, using both Somali and English. The co‐researchers analysed the data together using thematic analysis.

### 2.2 Findings

Four major themes were identified: ‘My child is different’, ‘Perceptions of autism’, ‘Navigating the system’ and ‘Support’. These are reported in full elsewhere.^16^, ^17^ In summary, the research identified the challenges faced by families in the Bristol Somali community in accessing support for their children with autism. There is no Somali word for autism making it hard to understand and accept. Existing cultural stigma related to mental health, challenging behaviour and disability reinforces families’ tendency to hide their child and to avoid seeking help early. Parents often feel isolated and do not engage with support services for their child. The findings highlight the importance of service providers understanding cultural views of autism and the need to raise awareness within the community, reduce stigma and provide support to encourage families not to delay seeking help for their children.

### 2.3 Dissemination

With agreement from members of AI, this co‐produced knowledge was shared widely with a range of audiences. Community theatre had demonstrated the power of communicating sensitive information to diverse audiences and so the team developed a joint presentation, which brought the research findings to life through extracts from ‘Yusuf can't talk’ (see Box 2). The research team in collaboration with ACTA theatre company gave a series of presentations at professional conferences, community events and to local health and social care partners, including Bristol city council. Audiences engaged with this format and many commented that the findings have resonance for other migrant and BME communities. Such feedback encouraged Nura to seek more ambitious channels of dissemination. She connected with local members of the Parliament of the United Kingdom of Great Britain and Northern Ireland, leading to an invitation to present to the All Party Parliamentary Group on Autism at the House of Commons. The combination of these activities was picked up by the media. Local and national radio and television coverage followed Women's hour,^18^ BBC and BBC World Service and Buzzfeed.^19^ All of this culminated in Nura’s TEDx talk, 'No More Us and Them ‐ Disrupting Attitudes to Autism'.^20^

Box 2 **Reaching out through theatre and research**

1 One of the first steps I took was to contact ACTA, a community theatre company. They agreed to work with myself and other mothers in AI to develop and perform a play; ‘Yusuf can't talk’. There were two objectives and outcomes of the play; exposing what life is like for families living with autism; and providing a picture for practitioners to understand cultural barriers. Research demonstrates that drama can be effective medium for communicating sensitive information. We performed the play 6 times in Bristol, once in Holland and later in other UK cities. The audiences were varied and the message reached many people who might not otherwise have heard and understood about autism in the Somali community.

As I met other families affected by autism in my local community, I increasingly learnt that my community are just one of several migrant groups among whom research has identified a higher prevalence of autism. I found out that Dheeraj Rai, a researcher at University of Bristol was involved with autism and migration research in Sweden. So, I contacted him requesting a meeting. Initially I asked him whether more research could be done to discover the reasons for such a high prevalence within the Somali community. Dheeraj suggested that together we apply to an open call for research ideas to the newly established NIHR CLAHRC West. He explained that this funding opportunity might not allow us to explore the reasons behind autism but that we may be able to do some work understanding of the difficulties that families in Bristol are facing to get support for their children with autism.

Dheeraj also suggested that a grant could support the dissemination of the play and the research. Together ACTA, AI and the University of Bristol applied for and were awarded funding from the Wellcome Trust to give a series of presentations.

### 2.4 Impact

As the co‐produced knowledge was disseminated more widely, professionals working with Somali families began to request more resources to increase their cultural understanding of autism, to refine and improve the delivery of services. Aware that policymakers, practitioners, community leaders and others could use the research findings to make change, the team explored ways to broaden the impact of their research, as advocated by proponents of Participatory Health Research (PHR).^21^ Considering how effective community theatre had been in awareness raising, the team decided that producing a film could reach multiple audiences and provide a lasting resource for on‐going use. A short film could be used to illustrate and bring to life the content of training whilst also being available for Somali families in areas not supported by AI. The agreed aims of the film were (1) to increase understanding and tackle stigma among Somali migrant communities and (2) improve awareness of culture‐specific issues in autism among health, education and social care professionals, trainees and policymakers. It will be embedded in existing training for all three sectors and will be freely available online for wider use. The film 'Overcoming Barriers; autism in the Somali community' was launched in April 2019^22^ (Box 3).

Box 3 **Final reflections**

1 Nura: Imagine being an outsider to the research world; a Somali mum of a child with autism. Imagine the point at which you realise that you became a researcher, familiar with research processes, ethical considerations, interviewing styles, data analysis and presenting research findings for different audiences. Imagine realising how things can change for you, for your child and for your community.

At times my dual roles as researcher and community worker conflicted, creating tension for me. I knew that some participants were not revealing the full extent of their difficulties. I had to contain my personal feelings at times to make sure that the interviews reflected a range of views. The positive response to the research showed me the power of research in giving more of a voice and raising awareness about autism in our community. This increased my motivation to conduct a PhD in this area. Furthermore, through‐out this process I immediately felt how important it is for participants having a role with the whole process of the research rather than getting data from them. I ensured that the AI families were informed and involved in making decisions at every step of the process. Seeing how some of our parents have bravely agreed to take part in the film, 'Overcoming barriers' is proof to me that things are changing in our community: we are not hiding our children with autism instead we are spreading information and understanding both to the professionals and to the Somali community. This partnership has shown my community that research can be co‐produced with them and can help to begin making changes for them.

## 3 PROCESS OF CO‐PRODUCTION

It is acknowledged that a diversity of approaches to co‐production exists^23^, ^24^ leading to various ways of measuring its value or impact. In order to reflect on their experiences, the co‐researchers refer to recently defined principles and key features to guide co‐production in health research. These are drawn from NIHR INVOLVE, an organization which supports active public involvement in the NHS, public health and social care research^7^ and from Roper and Grey,^11^ who define core principles of co‐production for mental health research (see Table 1). The current authors have grouped these as (1) establishing effective partnerships and building relationships between individuals and organizations^7^, ^11^; (2) maintaining relationships through reciprocity, power‐sharing, inclusion of all perspectives and skills, and valuing knowledge of all partners^7^; and (3) developing skills and capacity and opportunities for personal growth.^6^ These principles are reflected upon using first‐person narrative insights into Nura and Fiona’s subjective experience of coproduction.

**Table 1:** The authors’ core principles of co‐production: drawn from key principles defined by INVOLVE^7^ & Roper and Grey^11^

Authors	INVOLVE^7^	Roper and Grey^11^
1. Establishing effective partnerships	(1) Sharing of power: the research is jointly owned and people work together to achieve a joint understanding (2) Including all perspectives and skills: make sure the research team includes all those who can make a contribution (3) Respecting and valuing the knowledge of all those working together on the research: everyone is of equal importance (4) Reciprocity: everybody benefits from working together (5) Building and maintaining relationships: an emphasis on relationships is key to sharing power. There needs to be joint understanding and consensus and clarity over roles and responsibilities. It is also important to value people and unlock their potential.	(1) Consumers are partners from the outset: consumers are involved in setting the priorities and agenda and making decisions from the very beginning
2a. Building and maintaining relationships with organizations and communities 2b. Developing individual relationships through flexibility and reflection 2a and 2b through: Reciprocity Power‐sharing Inclusion of all perspectives and skills Valuing knowledge of all partners		(2) Power differentials are acknowledged, explored and addressed: Co‐production means that the more powerful partners relinquish power and support empowering environments for others. Using a co‐production methodology means the balance of power is challenged and consumers can exert influence
3. Developing skills, capacity and opportunities for personal growth		(3) Consumer leadership and capacity are developed: Co‐production is a mechanism for learning and developing knowledge. A genuine partnership builds the capacity and harnesses the knowledge and skills of everyone involved

**1. Establishing effective partnerships**

Guidelines indicate the importance of consumers being partners from the outset.^6^ In this project, the partnership was initiated by Nura, on behalf of AI, seeking collaboration with the University through Dheeraj. Dheeraj's knowledge of sources of research funding enabled them to access funding from NIHR CLAHRC West, to conduct qualitative research and from the Wellcome Trust to disseminate the community theatre work with ACTA Theatre. Through sharing their academic knowledge and lived experience of autism in migrant communities, the individuals representing their organizations began to establish working relationships.

Nura and Dheeraj’s idea represented a unique opportunity to co‐produce research with members of the Somali community, the second largest migrant group in Bristol. Sabi, leader of CLAHRC West's Ethnography team, had experience of research with immigrant communities in Birmingham and was therefore well placed to guide the development of such a project. At this stage, the partners saw the opportunity to collaborate, sharing knowledge, skills and experiences for mutual benefit. Through initial meetings, a research agreement document was written which formalized the contract between the newly established partnership. At this early stage, the team negotiated the research question, which has been highlighted as a potential challenge where priorities and values differ.^1^ At this stage, Nura and Fiona both reflected on their sense of being outsiders;

Nura: *I had little experience of research when this journey began and needed to assert myself in these early meetings. I was glad that the problems facing our community were being taken seriously by the researchers but as we discussed the research question, I kept stating that the focus should be on families’ access to services. The other members of the team agreed and together we planned an interview schedule that would explore this issue. The practical task of translating recruitment materials was the first step in working with Fiona on this project.*

Fiona: *Despite my extensive experience in qualitative research, I was initially unsure how the Somali parents might perceive me and whether they would feel comfortable in telling me their stories. On meeting Nura, I felt reassured that her lived experience would be critical to bridge the gap between myself, an unknown researcher, and Somali parents affected by autism.*

**2a. Building and maintaining relationships with organizations and communities**

As familiarity grew between Nura and the research team, it was essential that the wider community was fully involved in the proposed research. As noted by Kothari et al,^24^ this required commitment to collaboration, communication, rapport building and negotiation. Early ideas and plans for the research were discussed at community meetings with Somali parents. Sabi attended these meetings to familiarize the families with research and to explore their views about a research partnership. Nura provided ‘cultural brokerage’ between the study team and the local Somali community, and mediated between potential participants’ enthusiasm for quick action and improvement, and the slower pace required for research processes. The concept of research is not always well understood in non‐western communities.^25^ Initially, some of the Somali parents thought this research could lead to finding a ‘treatment’ or ‘cure’. Nura and Sabi consistently clarified that the aims of the research were not to seek a cure for autism but to help understand the experiences and needs of families affected by autism. Nura noted that this uncertainty cropped up many times throughout the life cycle of the project and at each stage she had to find ways to explain the nature and purpose of the research: Nura reflected: *There were times participants asked what would happen to their interview and what it means for them. As a member of their community they were seeking my reassurance that they were in safe hands. I had to offer more support and spend time describing the meaning of research. Some of the research language could be difficult to explain, for example the word ‘consent’ could not be exactly translated.*

Fiona noted that because she could not speak Somali, it was challenging to develop trust and rapport with participants: *their non‐verbal cues were not always easy for me to read and this maintained my sense of being an outsider. I relied on Nura to maintain rapport which felt uncomfortable at times.*

It has been acknowledged that ‘the power and privilege conferred on researchers by their university affiliations may potentially affect collaborative processes with other stakeholders and communities’.^12^ In order to redress the balance of power, the team attempted to create a sense of ownership of the research and its outputs among the members of AI, via regular community meetings throughout the project. These were structured to elicit feedback which was then built into the project. Before starting the dissemination phase, Fiona and Nura together presented the findings of the research at a well‐attended community meeting. It stimulated much debate and elicited feedback that the themes made sense to other families. As plans developed for further impact work, informal discussions were held at AI to ascertain the priorities and views of Somali families who are affected by autism. The co‐researchers believe that this was a crucial part of maintaining communication and demonstrating that the research was not merely an opportunity to ‘take knowledge’ but could give something of value back to their organization to help increase understanding and tackle stigma about autism in the Somali community. At a meeting to discuss the film, mothers said that they wanted the film to include the voices of professionals who work with their children, in order to get some answers for the questions that they have.

While the concept of co‐production promotes equal partnership between professionals and citizens, this may be difficult to achieve or measure. In this study, equal power meant valuing experiential knowledge^26^ and actively sharing decision making^1^ at each stage of the project, from formulating the research question to agreeing recommendations and negotiating on‐going impact work.

**2b. Developing individual relationships through flexibility and reflection**

Successful co‐production requires specific personal qualities in key contributors, such as openness, tolerance and flexibility.^6^ Fiona reflected: ‘We relied on each other's insider expertise and knowledge of research and Somali culture to negotiate the phases of research. In particular, informed consent was challenging for Nura to explain to participants, requiring time and patience. In the data collection stage, through the process of bilingual co‐interviewing, which required openness, trust and continual reflection, our relationship as co‐researchers was cemented’.

When collecting data, it was of key importance that participants had the opportunity to tell their stories in their preferred language.^18^ Through a combination of Fiona’s experience of qualitative interviewing and Nura’s interpreting skills, the co‐researchers were able to successfully conduct the research interviews in English and Somali. The experience of bilingual co‐interviewing enhanced the working relationship between Fiona and Nura, bringing insight into each other's worlds. As participants switched between speaking English and Somali, the co‐interviewers had to be flexible in their roles and Nura negotiated the tasks of interpreting questions, phrasing questions in a culturally sensitive way and prompting for elaboration. Successful coproduction is predicated on co‐researchers coming together frequently to reflect on the research process^7^ and Nura and Fiona regularly reflected on their own interviewing techniques, as well as the experience for the participants. This enhanced trust and understanding between the co‐researchers, particularly when the interview had been emotionally charged, and helped them to refine their interview skills. The importance of ‘phases of reflection and action’ is highlighted in a study of co‐inquiry action research (CAR) between community and university partners involved in a research collaboration.^4^

The time and effort taken to invest in and manage relationships have been highlighted as a cost of co‐production.^1^ Negotiating and renegotiating their identities as insiders and outsiders also had some personal costs for the co‐researchers. Throughout project, Nura and Fiona had to challenge themselves regularly to step outside the comfort of their own identities and roles. Nura attended several academic and professional conferences which she initially experienced as an ‘outsider’. When attending community events, Fiona had to tolerate her own discomfort of the language barrier and lack of understanding. These costs were offset by the benefits of personal and professional growth, as well as to the project as a whole.

**3. Developing skills and capacity through opportunities for personal growth**

Both Nura and Fiona benefited from increased expertise and capacity in their skills as qualitative researchers. Before starting the research, Nura completed a course in qualitative research skills, which increased her understanding of and involvement in the research process. Sharing the process of systematic data analysis was a learning curve for Nura and her research skills were supported by Fiona. Nura reflected that: ‘Fiona was a research mentor for me, whilst I was a mentor for her in understanding the community, bringing greater depth to her analysis’. Presenting the research findings required flexibility as Fiona and Nura negotiated a structure to the presentations that played to both of their strengths and ensured they were presenting the research as equals. Nura’s increased research experience enabled her to take up posts to conduct several further research projects linked to Somali and BME community well‐being research where the combination of cultural understanding and research experience was a distinct asset. It further motivated and empowered her to pursue further her dream of undertaking a PhD. From an organizational perspective, the capacity of AI grew significantly during the lifespan of the research, community theatre and on‐going impact work. Growing interest in the organization enabled AI to appoint an advisory board. This will be discussed in the subsequent section.

## 4 IMPACT

In line with quality criteria for Participatory Health Research (PHR), this study produced knowledge which was ‘local, collective, co‐created’^21^ The findings supported previous research that immigrant populations require appropriate help and support in relation to autism services,^14^ not least because early diagnosis and intervention led to improved outcomes for their children.^27^ In Bristol, the Somali community are the second largest migrant group and more than 80 Somali families are known to have one or more children with autism.

Participatory Health Research advocates that co‐produced knowledge is accessible to multiple audiences, over and above academic communities^21^ and the knowledge for this study had relevance for Somali communities in other cities and countries, who have less well‐established networks of support, as well as for other BME groups who may face similar cultural challenges when seeking support for autism. The need to improve awareness, reduce stigma and provide support to encourage families not to delay seeking help for their children was of key importance.

It also had relevance for policymakers, practitioners and others who could use the information to make change.^21^ As the co‐researchers shared the study findings, professionals working with Somali families began to request more resources, to increase their cultural understanding of autism, to refine and to improve the delivery of services. This led to a number of new synergies, as the team strove for a broad impact to bring about change through social learning.^12^, ^21^

In the second part of the paper, the co‐authors map the micro‐ to macrolevels of impact that grew from this co‐produced knowledge, using a ‘social model of impact’ and framework which aims to ‘capture multi‐layered and potentially transformative impacts of co‐produced research’.^12^

### 4.1 Micro: Individual

Nura reflected on the transition from her ‘weak public voice’, prior to the research collaboration to a ‘strong public voice’^28^ after its dissemination (most aptly illustrated through her TEDx talk^20^). Nura’s lived experience as a Somali mother of a child with autism meant that to her the research findings were not necessarily ‘new and unique knowledge’.^8^ However, she noted the distinct difference in the way that organizations responded to her requests for support once the research had been published and widely shared. She believed that it gave credibility and strength to the mission of AI to improve support for and cultural understanding about Somali families affected by autism. Nura reflected: *Although it was challenging and at times even intimidating, disseminating the research findings enabled me to access a wide audience to share the barriers experienced by Somali families with autism. It allowed me to combine my lived and learned experience (living with autism, social work, research experience and MSc) for social change. My role as an insider carried the weight of the voices of the many AI families that I work with. As I stood in front of so many different practitioners, my method of disseminating was to deliver with both emotions and evidence.*

For the research team, the experience also had individual level impact in increasing skills and expertise in coproducing research with an under‐served community, using bilingual interviewing.

### 4.2 Micro: Group

The success of this initially small‐scale research project demonstrated to all partners the potential for future collaboration, increasing trust and willingness to work together in the future. The co‐produced research led to on‐going collaborative work between the initial partners (AI, CLAHRC West, ACTA and University of Bristol) and new partners (Therapeutic Media) to produce resources for greater impact. The film Overcoming Barriers: autism in the Somali community^22^ is a tangible example of how successful co‐production in research can lead to fruitful working relationships for on‐going impact.

### 4.3 Meso: Organization level

AI has greatly increased in capacity since the research and allied work began, from 50 families in 2015 to more than 80 families by 2019. Much of this is due to the increased awareness among the Somali community about autism and the availability of support through AI. Tangible benefits have also been realized for the academic organizations who have published a number of peer‐reviewed papers and have had the opportunity to share of the co‐produced knowledge with a variety of local and national stakeholders. The film^22^ is being embedded into training for health, education and social care professionals, trainees and policymakers to improve awareness among service providers of culture‐specific issues in autism.

Beckett et al^12^ suggest that macrolevel impact can be achieved through, ‘brokering relationships and engaging with opportunities that arise from co‐produced work’. Since disseminating the research findings, opportunities have led to the formation of partnerships and synergies between AI and a range of health, social care and education providers.

#### 4.3.1 Health

Healthwatch Bristol worked with AI to produce a report,^29^ which been widely shared and has been used alongside the research to shape services for Somali families affected by autism. The recommendations of this report led to The People's Health Trust funding 36 workshops over a period of a year for Somali families. The workshops made up of three sessions a month, focussed on topics including ‘what is autism’, ‘sensory disorder’, ‘behaviour management’ and ‘types of communication’. AI also started a wider project with Barnardos, Sirona and the NHS Community Children's Health Partnership to explore the barriers that are preventing BAME (Black, Asian Minority Ethnic) communities from accessing mainstream health services.

#### 4.3.2 Social care

The Bristol City Council Autism Team worked with AI, after noting that few Somali families attended their workshops. Together they developed specific workshops for Somali families, which were well attended by 17 families. AI and The West of England Centre for Inclusive Living (WECIL) started a drop‐in session to support families to complete paperwork to access the disability living allowance. This was initiated by a local councillor who had read the research papers and the Healthwatch report and who felt this could reduce some of barriers faced by the Somali community.

#### 4.3.3 Education

AI now advises and supports several schools in the Bristol area, including collaborations between six local schools to improve their work with Somali families affected by autism. AI and Venturers Academy have trialled a successful holiday camp and parent workshops over the summer period. The workshops were well attended and helped to increase awareness of their child's longer term independence. This has attracted wide interest and was covered by the BBC.

The variety of synergies and outcomes from this project illustrate that co‐produced knowledge can be disruptive, leading to transformative social outcomes.^30^

### 4.4 Macro: Societal

Beckett et al propose that co‐produced knowledge can be ‘transformative at a broader macroscale where co‐produced research combines with other interventions, wider policies or practice priorities to create dynamic synergies’.^7^ While it is difficult to assess the macroimpact of co‐produced knowledge, a facilitator to achieving impact at a societal level maybe achieved through presenting co‐produced knowledge in accessible and creative formats.^12^ The use of both community theatre and film is examples of how this co‐produced knowledge reached multiple audiences and disseminated knowledge widely in a lasting format. Since its launch, the film Overcoming Barriers^22^ has been shared internationally, viewed more than 150 000 times, and is generating discussion and debate.

## 5 CONCLUSIONS

This paper presents the reflections of co‐researchers about their experiences of a co‐production journey, as insiders and outsiders, stepping in and out of each other's worlds. The sharing of skills, knowledge and power was central to this process and was achieved through the development and maintenance of relationships, reciprocity, flexibility and continuous reflection. Through shared experiences and learning, skills and capacity were built both for individuals and organizations. The co‐produced knowledge was mobilized in creative and accessible ways, through theatre, film, media and TEDx talks, reaching diverse audiences, locally, nationally and internationally. This was only achieved through the development of partnerships with numerous organizations. In turn, this sparked synergy with providers in health, social care and education. This project demonstrates how real opportunities for all partners to be equitably involved to the maximum degree possible throughout the research process can affect social change.^21^ Ultimately, this is a story of how co‐produced research can become a catalyst for impact that is dynamic and complex achieving multi‐layered impact.

In summary, the co‐researchers identify key challenges for consideration, as well as factors which contributed to this co‐produced knowledge and the on‐going impact:

Challenges to co‐production:

- The investment required by community research partners to explain and build trust in the research process
- Language barriers, cultural understandings and lack of shared concepts such as scientific research and consent, which can affect trust and rapport between researchers and community members
- The time and effort required for co‐researchers to step outside their comfort zones into each other's worlds.

Factors supporting co‐production:

- The involvement of at least one person who is willing and able to advocate for a community group and to bridge the gap between research institutions and community organization
- Building and maintaining trust between key players in the coproduction process. This can be achieved through continual reflection, appreciation of and sharing knowledge and expertise, and commitment to flexibility within roles
- Willingness to engage in creative forms of knowledge sharing in order to reach diverse audiences, such as community theatre and film
- Willingness to harness opportunities to collaborate with organizations who have capacity to take the research findings and key messages and implement them into social change.

## CONFLICT OF INTEREST

None.
